# Genome-Wide Re-Identification and Analysis of CrRLK1Ls in Tomato

**DOI:** 10.3390/ijms24043142

**Published:** 2023-02-05

**Authors:** Wenpeng Ma, Xin Liu, Kai Chen, Xinlong Yu, Dongchao Ji

**Affiliations:** 1 College of Agricultural Engineering and Food Science, Shandong University of Technology, Zibo 255049, China; 2 School of Life Sciences and Medicine, Shandong University of Technology, Zibo 255049, China; 3 Key Laboratory of Plant Resources, Institute of Botany, Innovative Academy of Seed Design, Chinese Academy of Sciences, Beijing 100093, China; 4 University of Chinese Academy of Sciences, Beijing 100049, China

**Keywords:** CrRLK1L, *Solanum lycopersicum*, gene family analysis, genome-wide analysis

## Abstract

The *Catharanthus roseus* receptor-like kinase 1-like (CrRLK1L), which is a vital member of the plant receptor-like kinase family, plays versatile roles in plant growth, development, and stress response. Although the primary screening of tomato CrRLK1Ls has been reported previously, our knowledge of these proteins is still scarce. Using the latest genomic data annotations, a genome-wide re-identification and analysis of the CrRLK1Ls in tomatoes were conducted. In this study, 24 CrRLK1L members were identified in tomatoes and researched further. Subsequent gene structures, protein domains, Western blot analyses, and subcellular localization analyses all confirmed the accuracy of the newly identified SlCrRLK1L members. Phylogenetic analyses showed that the identified SlCrRLK1L proteins had homologs in *Arabidopsis*. Evolutionary analysis indicated that two pairs of the *SlCrRLK1L* genes had predicted segmental duplication events. Expression profiling analyses demonstrated that the *SlCrRLK1L* genes were expressed in various tissues, and most of them were up- or down-regulated by bacteria and PAMP treatments. Together, these results will lay the foundation for elaborating the biological roles of SlCrRLK1Ls in tomato growth, development, and stress response.

## 1. Introduction

As a crucial member of signal transduction, receptor-like kinases (RLKs) constitute the largest receptor family in plants and play a significant role in plant growth, development, stress, and pathogen response [1,2]. According to their diverse extracellular domains, plant RLKs can be mainly divided into the following: the S-domain, the wall-associated kinase domain, the legume lectin domain, the CRINKLY4 domain, the malectin-like (CrRLK1L) domain, the malectin-like leucine-rich repeat domain, the leucine-rich repeat malectin domain, the cysteine-rich repeat domain, the leucine-rich repeat (LRR) domain, the lysin motif domain, the pro-rich/extension domain, and the calcium-dependent lectin domain RLK family [3]. To the interest of many researchers, the plant-specific CrRLK1L protein kinases were firstly identified in Madagascar periwinkle and have since been found to exist in a variety of plant species [2,4,5]. Traditionally, CrRLK1Ls possess the following three conserved domains: the malectin-like domain, the transmembrane helix domain, and the kinase domain [5]. Some of the CrRLK1L members have been functionally identified, including FERONIA (FER), ANXUR1/2 (ANX1/2), THESEUS1 (THE1), BUDDHA’S PAPER SEAL1/2 (BUPS1/2), and HERCULES1 (HERK1).

The CrRLK1L family members are involved in a wide range of biological process regulations, including male–female gametophyte recognition, cell expansion, hormone signaling, energy production, stress tolerance, and host–pathogen interactions [2,6,7,8,9,10,11]. AtFER, which was originally identified from a pollen tube mutant, has become the most extensively investigated CrRLK1L protein in *Arabidopsis* [5,12,13]. AtFER takes part in different hormone signals that contain auxin, ethylene, brassinosteroid (BR), abscisic acid (ABA), and jasmonic acid (JA) [6,14,15,16,17]. Moreover, AtFER acts as a receptor for RALF1 (rapid alkalinization factor 1), RALF17, RALF23, RALF32, and RALF33 to regulate development and biotic/abiotic stress responses [18,19,20]. As for other *Arabidopsis* CrRLK1L members, AtTHE1 and AtHERK1 have been reported to regulate cell elongation [21,22], AtANX1/2 and AtBUPS1/2 participate in pollen tube growth regulation [11,23], and MEDOS1 (MDS1), MDS2, MDS3, and MDS4 have been reported to be involved in metal ion stress responses [24]. In addition, AtFER is involved in host–pathogen interactions, including *Golovinomyces* (*syn*. *Erysiphe*) *orontii* and *Pseudomonas syringae* pv. tomato DC3000 responses [17,25]. Several CrRLK1L members have also been functionally identified in other species. In rice, it has been reported that OsFLR1 (*Oryza sativa* FERONIA-like receptor1) and OsFLR2 (also named DRUS1 and DRUS2) are essential for maintaining architecture, reproduction, and seed yield [26,27]. Moreover, the ruptured pollen tube (RUPO) regulates the growth and integrity of pollen tubes [28]. Apple MdFERL1 (*Malus domestica* FERONIA-like1), MdFERL6, and tomato SlFERL (*Solanum lycopersicum* FERONIA-like) are involved in fruit ripening [29,30]. In soybean, GmLMM1 (*Glycine max* lesion mimic mutant1) regulates cell death and PTI (pattern-triggered immunity) processes, responding to bacterial and oomycete pathogen infections [31]. In pears (*Pyrus bretchneideri*), PbrCrRLK1L3 and PbrCrRLK1L26 take part in the pollen tube rupture process and growth [32]. Additionally, *Chenopodium quinoa* CqFER, soybean GmCrRLK1L20, and tobacco NtCrRLK1L47 have been reported to be involved in salt stress responses [33,34,35].

The tomato (*Solanum lycopersicum*), as an economically important fleshy fruit crop, is widely accepted as a model species for studying the developmental and postharvest biology of horticultural crops. The genome sequencing and annotations of tomatoes were completed for the first time in 2012 [36]. Based on this, a previous genome-wide receptor-like kinase (RLK) study found 23 CrRLK1L protein kinase subfamily members in the tomato genome, but it did not analyze the gene structure, protein motifs, phylogeny, subcellular localization, and gene expression of these proteins [37]. Over the past decade, with the increasing ability of technology, the annotation of the tomato genome has become more sophisticated and accurate. Therefore, it is necessary to re-identify and analyze the CrRLK1L protein kinases in tomatoes. In this study, taking advantage of the state-of-the-art and well-annotated tomato genome and protein database, genome-wide research of the CrRLK1L protein families was performed. As a result, 24 CrRLK1L protein kinase candidates were identified in the tomato genome. Further analysis showed the phylogenetic relationship, physicochemical properties, gene structure, subcellular localization, conserved protein domains, predicted motifs, and gene expression pattern of this family. It will give us new insight into the tomato CrRLK1L family and help us reveal the function of these proteins in the future.

## 2. Results

### 2.1. Identification of Tomato CrRLK1L Protein Kinases

**Figure 1:**
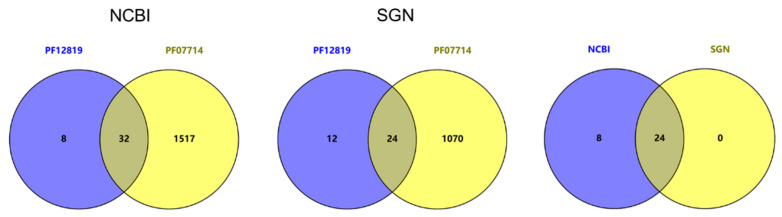
Identification of tomato CrRLK1Ls in the NCBI and SGN databases. The predicted numbers of tomato CrRLK1Ls in the NCBI and SGN databases are shown. PF12819: Malectin-like; PF07714: PK-Tyr-Ser-Thr.

**Figure 2:**
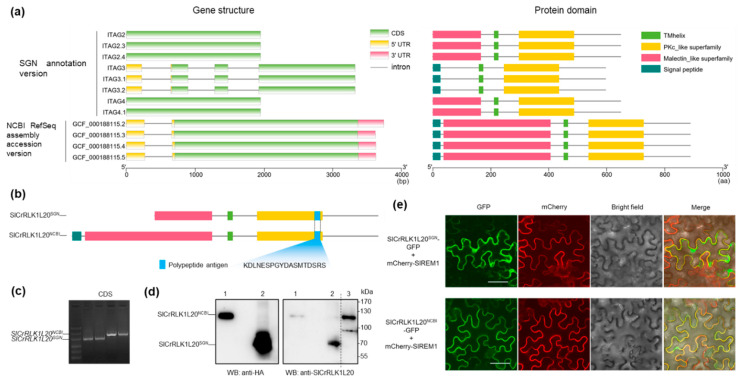
The accuracy of the tomato CrRLK1L annotations in NCBI was higher than that in SGN. (**a**) The different versions of the gene structure and protein domain of SlCrRLK1L20 in the NCBI and SGN databases. The data were extracted from NCBI and SGN and then analyzed for visualization; detailed information can be found in Table S2. (**b**) Polypeptide antigen location in SlCrRLK1L20^NCBI^ and SlCrRLK1L20^SGN^. (**c**) Amplification of the *SlCrRLK1L20^NCBI^* and *SlCrRLK1L20^SGN^* CDSs. (**d**) Western blot analysis of SlCrRLK1L20^NCBI^ and SlCrRLK1L20^SGN^. 1: *N. benthamiana* leaves transiently expressing CaMV35S::SlCrRLK1L20^NCBI^-HA; 2: *N. benthamiana* leaves transiently expressing CaMV35S::SlCrRLK1L20^SGN^-HA; 3: *S. lycopersicum* fruit. The uncropped Western blot gel image can be found in Figure S2. (**e**) Confocal analysis of SlCrRLK1L20^NCBI^ and SlCrRLK1L20^SGN^ subcellular localization. Bars = 50 μm.

All CrRLK1L protein kinases consist of a malectin-like domain and a kinase domain. *Arabidopsis* CrRLK1L protein kinase sequences were submitted to the Pfam database, and two conserved domains (Pfam: PF12819 and PF07714) were acquired. Based on these criteria, the two conserved domains served as queries to screen the tomato protein databases in the National Center for Biotechnology Information (NCBI) and the Sol Genomics Network (SGN). As shown in Figure 1, 32 and 24 *Cr*RLK1L protein kinase candidates were identified in NCBI and SGN, respectively. As a result, 24 tomato *Cr*RLK1Ls were matched to both databases and named from SlCrRLK1L1 to SlCrRLK1L24 according to the location of the chromosomes (Table 1). Meanwhile, there were differences in the annotation of ten proteins in the NCBI and SGN databases (Figure S1; Table 1; Table S1). One of them was chosen to verify the accuracy. We analyzed the gene structure and protein domain of SlCrRLK1L20 from the SGN annotation versions (from ITAG2 to ITAG4.1) and the NCBI RefSeq assembly accession versions (from GCF_000188115.2 to GCF_000188155.5) and found that the NCBI RefSeq had complete UTR, CDS, intron, signal peptide, transmembrane helix, malectin-like, and protein kinase descriptions (Figure 2a). Moreover, the *SlCrRLK1L20* gene sequences from SGN were all included in NCBI. Then, a unique polypeptide was used as an antigen to produce a SlCrRLK1L20 antibody, which could detect SlCrRLK1L20^NCBI^ (NCBI GCF_000188115.5, the newest annotation protein) and SlCrRLK1L20^SGN^ (SGN ITAG4.1, the newest annotation protein) simultaneously (Figure 2b). The *SlCrRLK1L20^NCBI^* and *SlCrRLK1L20^SGN^* CDSs were amplified by PCR (Figure 2c) and used to construct a plant expression vector. A Western blot assay showed that SlCrRLK1L20^NCBI^ was detected by the anti-SlCrRLK1L20 antibody in the tomato but SlCrRLK1L20^SGN^ was not (Figure 2d). At the same time, the subcellular localization of SlCrRLK1L20^NCBI^ and SlCrRLK1L20^SGN^ was conducted by confocal analysis. *Solanum lycopersicum* REMORIN1 (SlREM1) was identified as a plasma membrane-labeled protein in previous research [38]. As shown in Figure 2e, SlCrRLK1L20^NCBI^-GFP was co-localized with SlREM1 at the plasma membrane, while SlCrRLK1L20^SGN^ was not. The above results revealed that the annotation of SlCrRLK1L20 from NCBI was more accurate than SGN. Therefore, the subsequent related research was mainly based on the NCBI database.

### 2.2. Phylogenetic Analysis of the Tomato CrRLK1L Protein Kinases

**Figure 3:**
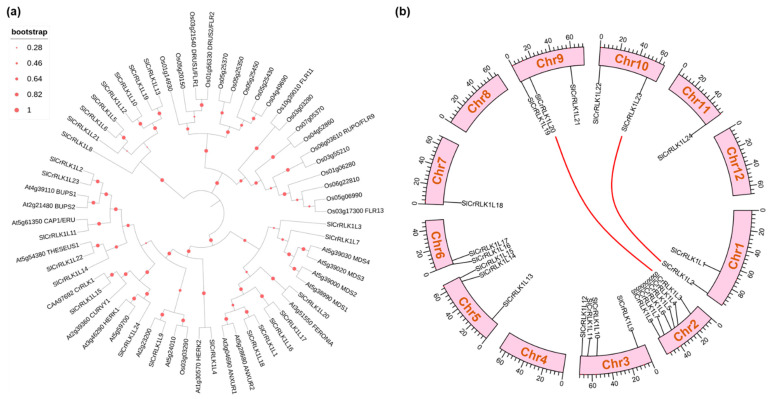
Evolutionary analysis of SlCrRLK1Ls. (**a**) Phylogenetic analysis of CrRLK1Ls in tomato, *Arabidopsis*, rice, and Madagascar periwinkle. (**b**) Chromosome position and collinearity of *SlCrRLK1Ls*. The collinearity relationship is marked by red lines. Unit: Mb.

In order to explore the relationship between *Arabidopsis*, rice, Madagascar periwinkle, and tomato CrRLK1L protein kinases, a phylogenetic analysis using the whole CrRLK1L amino acid from the above species was conducted (Figure 3a). The results revealed that most of the tomato SlCrRLK1Ls had several homologous *Arabidopsis* members but only one homologous member in rice, indicating that the evolution in different species is independent. *Arabidopsis* and tomato had a much closer evolutionary relationship than rice (Figure 3a). Several CrRLK1L proteins, including AtFERONIA, AtCAP1/ERU, AtCURVY1, AtTHESEUS1, AtANXUR1/2, AtBUPS1/2, and AtHERK1, have been well characterized in *Arabidopsis*. In tomato, one copy of SlFERONIA (SlCrRLK1L20), SlCAP1/ERU (SlCrRLK1L11), and SlCURVY1 (SlRLK1L15) and two copies of SlTHESEUS1 (SlCrRLK1L22/SlCrRLK1L14), SlANXUR1/2 (SlCrRLK1L1/SlCrRLK1L18), SlBUPS1/2 (SlCrRLK1L2/SlCrRLK1L23), and SlHERK1/2 (SlCrRLK1L24/SlCrRLK1L4), were identified.

### 2.3. Tomato CrRLK1L Gene Locations and Duplication on Tomato Chromosome

To better understand the relationship between tomato *CrRLK1L* genes, the chromosomal distribution and collinearity of these genes were analyzed by TBtools. The results were as follows: The tomato *CrRLK1L* genes were distributed on chromosomes 1 to 3, 5 to 7, and 9 to 11, and not distributed on chromosomes 4, 8, and 12 (Figure 3b). Chromosome 2 had the largest number of *SlCrRLK1L* genes, and chromosomes 7 and 11 had only one *SlCrRLK1L* gene (Figure 3b). Segmental duplication played an important role in the gene family expansion. During this study, one-step MCScanX was used to reveal the collinearity of the *SlCrRLK1L* genes. As shown in Figure 3b, there was a collinearity relationship between *SlCrRLK1L2*, *SlCrRLK1L23, SlCrRLK1L3*, and *SlCrRLK1L20*, which showed duplication events of these genes.

### 2.4. Tomato CrRLK1L Protein Domain and Gene Structure

**Figure 4:**
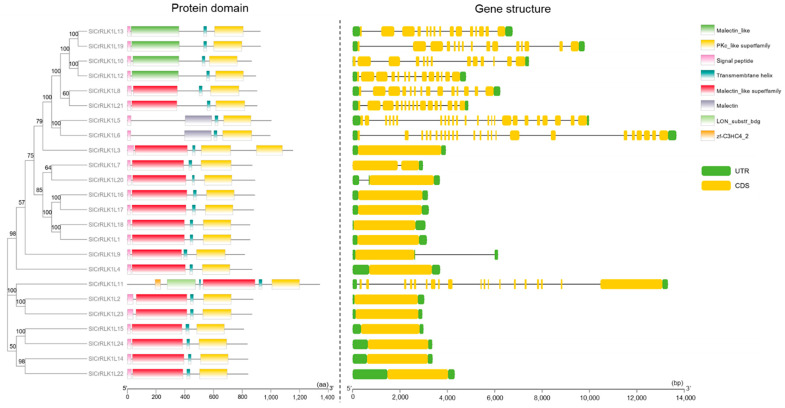
Protein domains and gene structures of SlCrRLK1Ls. The signal peptides, malectin-like domains, transmembrane helices, kinase domains, UTRs, and CDSs are marked in different colors.

In order to further confirm the SlCrRLK1L proteins, conserved domain detection was carried out. All of the sequences were submitted to the NCBI Batch CD-Search to search for common domains. As a result, the malectin domain, the malectin-like domain, and the PKc-like domain were verified (Figure 4). At the same time, DeepTMHMM (https://dtu.biolib.com/DeepTMHMM) (accessed on 3 September 2022) was used to detect the signal peptide and transmembrane helix of SlCrRLK1Ls. As shown in Figure 4, Tables S3 and S4, all of the SlCrRLK1L proteins held one signal peptide and one transmembrane helix, except for SlCrRLK1L11. SlCrRLK1L11 had two transmembrane helices and no signal peptides.

As for the gene structure, 13 out of the 24 *SlCrRLK1L* genes possessed continuous CDSs, and 11 *SlCrRLK1L* genes had no introns (Figure 4). A total of 4 of the *SlCrRLK1L* members (*SlCrRLK1L1*, *7*, *9*, and *20*) had only one intron, while the other 9 members (*SlCrRLK1L5*, *6*, *8*, *10*, *11*, *12*, *13*, *19*, and *21*) had multiple introns.

### 2.5. Prediction of SlCrRLK1L Conserved Protein Motifs

**Figure 5:**
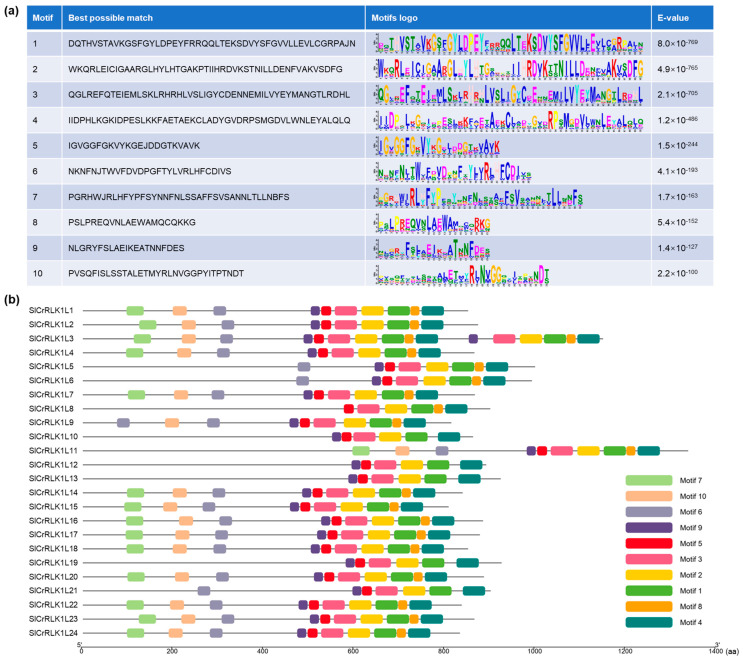
Predicted conserved motifs of SlCrRLK1Ls. (**a**) The best possible match sequences and motif logos are listed here. (**b**) Motif positions on the SlCrRLK1Ls.

The SlCrRLK1L conserved protein motifs were analyzed by MEME (https://meme-suite.org/meme/tools/meme) (accessed on 1 September 2022). In total, ten conserved motifs were acquired (Figure 5, motif one to ten); the amino acid numbers ranged from 21 to 50. Among them, motifs one to five could be found in all of the 24 members, while motif seven could only be found in 14 members (Figure 5). The similarities in the characteristic motifs between the SlCrRLK1L proteins may reflect functional similarities.

### 2.6. Subcellular Localization

Previous studies have found that most CrRLK1L proteins are localized in the plasma membrane. In this study, three assays were used to predict the subcellular localization of the SlCrRLK1L proteins. As shown in Table S3, almost all of the SlCrRLK1L proteins were predicted to localize in the plasma membrane, which was consistent with our SlCrRLK1L20 subcellular localization results. Meanwhile, the results obtained by different prediction methods were also different. The CELLO and MultiLoc2 shared most of their results, while the Plant-mPLoc Computation did not, owing to their various predicted algorithms. In addition, the signal peptide and transmembrane helix predictions of the SlCrRLK1L proteins further demonstrated the membrane localization of these proteins.

### 2.7. SlCrRLK1L Gene Promoter Analysis

**Figure 6:**
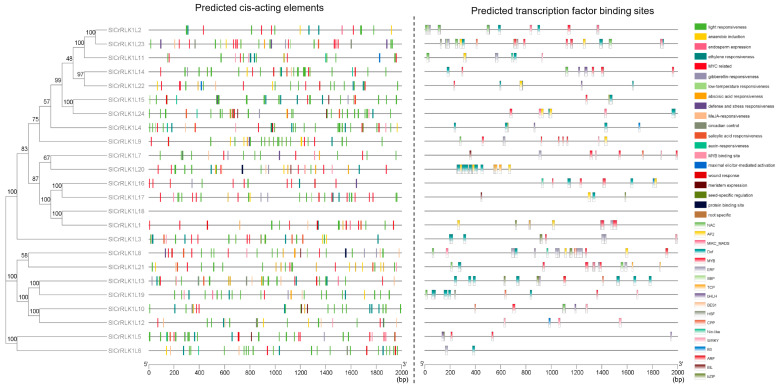
Predicted cis-acting elements and transcription factor binding sites of *SlCrRLK1L* promotors. The regions are represented by colored rectangles.

To better explore the putative functions in tomatoes, the *SlCrRLK1L* promoters were analyzed by PlantCARE (http://bioinformatics.psb.ugent.be/webtools/plantcare/html/) (accessed on 3 September 2022) and PlantTFDB (http://planttfdb.gao-lab.org/index.php) (accessed on 5 September 2022). The PlantCARE tool was used to detect the predicted cis-acting elements. As a result, 709 cis-acting elements were predicted in the *SlCrRLK1L* promoters, which were divided into 20 featured categories (Figure 6; Table S5). The predicted cis-acting elements were mainly related to light, low temperature, ethylene, gibberellin, abscisic acid (ABA), methyl jasmonate (MeJA), salicylic acid (SA), auxin, and wound responsiveness, suggesting that *SlCrRLK1L* may participate in hormone, stress, and defense responses. In addition, PlantTFDB was selected to predict transcription factor binding sites. As shown in Figure 6 and Table S6, 712 binding sites were identified in the *SlCrRLK1L* promoters; the represented sites were visualized and belonged to various types of transcription factors. Among them, NAC, AP2, MIKC-MADS, Dof, and MYB were the most abundant. However, there was no available data on the *SlCrRLK1L18* promoter because of incomplete sequencing or annotation of the genome.

### 2.8. SlCrRLK1L Gene Expression Pattern Analysis

**Figure 7:**
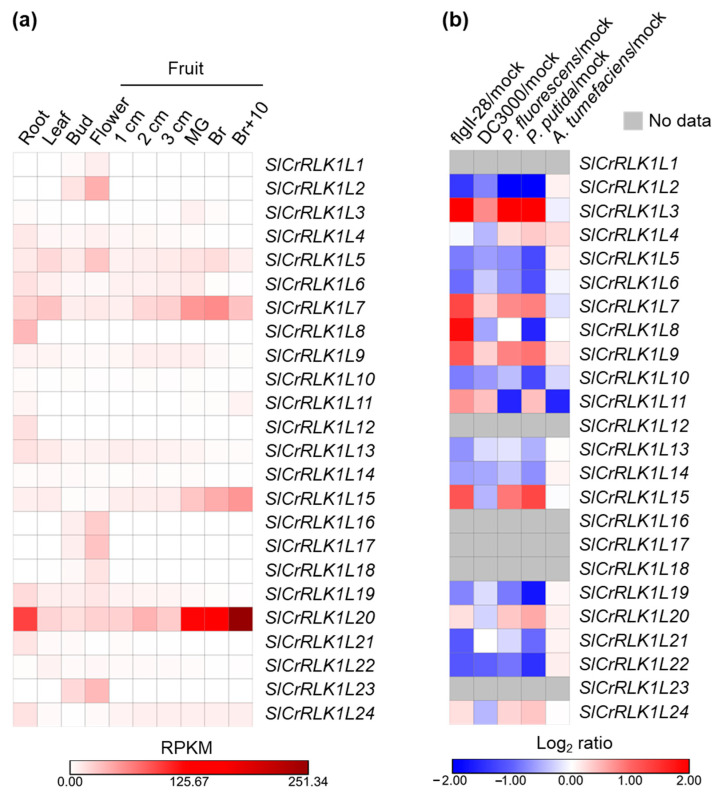
Expression pattern analysis of *SlCrRLK1Ls*. (**a**) Heatmap of *SlCrRLK1L* expressions in a variety of tissues. RPKM: reads per kilobase per million mapped reads. (**b**) Heatmap of *SlCrRLK1L* expressions in various bacteria and PAMP. No available data are represented by a gray color. All of the data were acquired from the SGN RNA-seq database.

It has been experimentally demonstrated that the *CrRLK1L* genes have tissue-specific expression patterns in *Arabidopsis*, tobacco, and apple [34,39,40]. To study the tissue-specific expression in tomato *CrRLK1L* genes, the expression profiles of all of the 24 *SlCrRLK1L* genes were examined in ten samples (root, leaf, bud, flower, from 1 cm to 3 cm of fruit, mature green fruit, breaker fruit, and breaker plus a 10-day fruit). The original RNA-seq data was extracted from the SGN tomato functional genomic database (SGN-TFGD, http://ted.bti.cornell.edu/cgi-bin/TFGD/digital/home.cgi) (accessed on 4 September 2022) [36,41]. As shown in Figure 7a, *SlCrRLK1L20* was dominantly expressed in all ten samples, especially in the roots and fruits. *SlCrRLK1L2*, *SlCrRLK1L5*, *SlCrRLK1L16*, *SlCrRLK1L17*, and *SlCrRLK1L23* were mainly expressed in the flowers. *SlCrRLK1L7* and *SlCrRLK1L15* had relatively high expression levels in the fruits, and *SlCrRLK1L7* held the highest expression level in the leaves as compared to the other genes. Compared with other tissues, *SlCrRLK1L8* and *SlCrRLK1L12* had relatively high expression levels in the roots. The other *SlCrRLK1L* genes held relatively low expression levels in all of the examined samples.

The promoter analysis of the *SlCrRLK1L* genes indicated that *SlCrRLK1L* might not only be involved in plant growth but also in defense responses. To explore this query, we calculated and compared the expression ratios of *SlCrRLK1Ls* treated with different bacteria and PAMP using the RNA-seq data from SGN-TFGD. The expression levels of the *SlCrRLK1L* genes changed with the different treatments, yet some of them possessed no available data (Figure 7b). When treated with flgII-28, a pathogen-associated molecular pattern (PAMP) founded in *Pseudomonas syringae* pv. tomato T1, *SlCrRLK1L3*, *7*, *8*, *9*, and *15* were up-regulated and *SlCrRLK1L2*, *21*, and *22* were down-regulated. Only *SlCrRLK1L22* and *SlCrRLK1L11* were significantly down-regulated by *Pseudomonas syringae* pv. tomato DC3000 or *Agrobacterium tumefaciens* infections. As for the *Pseudomonas fluorescens* and *Pseudomonas putida* treatments, *SlCrRLK1L2* was significantly down-regulated by *P. fluorescens* and *P. putida*, while *SlCrRLK1L3* was significantly up-regulated by these two bacteria.

## 3. Discussion

As an important member of the plant RLK family, CrRLK1Ls have been found in many species, including angiosperms (for example, *Arabidopsis*, rice, and apple), gymnosperms (*Picea abies*), and early diverging lineages (for example, the *Closterium peracerosum-strigosumlittorale* complex, *Marchantia polymorpha,* and *Physcomitrella patens*) (Table 2). However, the structural characteristics and functions of the tomato CrRLK1L gene family remain unclear. Based on this, we comprehensively analyzed the physicochemical properties, structural characteristics, and expression patterns of tomato CrRLK1Ls.

A previous study found that there were 23 CrRLK1L subfamily members in the tomato genome [37]. In this study, after sequence analysis, we used a new method to search the state-of-the-art and well-annotated tomato protein databases, and 24 SlCrRLK1Ls were re-identified. A comparison between the tomato CrRLK1L proteins in “this study” and a “previous study [37]” was also carried out. As shown in Figure S1, eight proteins were identified here for the first time. These proteins have not been identified previously, maybe due to the different analytical methods and genome annotations used. In addition, the annotations in SGN and NCBI had some differences. In short, some of the *SlCrRLK1L* gene structures from the SGN database lacked well-annotated UTRs and CDSs (Figure S1). To ensure the accuracy of the results, SlCrRLK1L20 was selected for further analysis. The results showed that NCBI had better annotations than SGN at this point (Figure 2). This situation is also present in other species. After the first identification, subsequent re-studies found that the number of CrRLK1Ls was different from that in previous studies of *Arabidopsis* and rice [27,57].

Homologous proteins often have similar functions. The phylogenetic analysis revealed that tomato CrRLK1Ls were closely related to *Arabidopsis*. Our homology search showed that 11 out of the 24 SlCrRLK1Ls had *Arabidopsis* homologs with known functions. It is speculated that these homologous genes may have evolved from a common ancestor, implying that they may have similar functions in some signaling pathways.

In *Arabidopsis*, rice, apple, strawberry, and soybean, CrRLK1Ls have been proven to be involved in development, fertility, environmental responses, and immunity [2]. Our cis-activating elements and transcription factor binding site analysis indicated that SlCrRLK1Ls may be involved in plant development, hormones, and environmental responses, such as auxin, ethylene, abscisic acid, wounds, light, and temperature (Figure 6), some of which were confirmed in *Arabidopsis* homologs, as illustrated above. Gene expressions were closely linked to their functions. During fruit ripening, *SlCrRLK1L20* showed a very high abundance of expression (Figure 7), which was consistent with its function in regulating fruit ripening [30]. Moreover, the expression pattern analysis suggested that SlCrRLK1Ls may participate in the response to bacterial infections. Upon treatment, *SlCrRLK1L2*, *3*, *8*, *11*, *15*, *19*, and *22* displayed relatively strong responses (Figure 7), indicating that these genes may be involved in plant–pathogen interactions. The functions of these SlCrRLK1L members need further exploration in the future.

In conclusion, we identified and analyzed the CrRLK1L family in tomato by bioinformatic, biochemical, and cell biology assays and provided a theoretical basis and guidance for further functional studies of these proteins.

## 4. Materials and Methods

### 4.1. Protein Identification and Phylogenetic Analysis

The genome sequence and annotations of tomatoes were downloaded from the National Center for Biotechnology Information (NCBI, https://www.ncbi.nlm.nih.gov/) (Bethesda, MD, USA, accessed on 31 August 2022) and the Sol Genomics Network (SGN, https://solgenomics.net/) (Ithaca, NY, USA, accessed on 30 August 2022) [36,58]. The *Arabidopsis*, rice, and *Catharanthus roseus* CrRLK1L protein sequences were downloaded from TAIR (https://www.arabidopsis.org/) (Newark, CA, USA, accessed on 20 April 2020) [59], EnsemblPlants (http://plants.ensembl.org/index.html) (Hinxton, United Kingdom, accessed on 20 April 2020) [60], and NCBI, according to their accession numbers. Firstly, the *Arabidopsis* CrRLK1L protein sequences were set as queries to Pfam (http://pfam.xfam.org/) (Hinxton, United Kingdom, accessed on 6 September 2022) [61] to identify their conserved domains. As a result, malectin-like (PF12819) and PK-Tyr-Ser-Thr (PF07714) HMM profiles were obtained and subjected to Simple HMM Search tools from TBtools v1.108 (Guangzhou, China) [62] to screen the tomato CrRLK1L protein candidates (sequence and domain scores, E-value < 0.05). The Venn image was illustrated by VENNY, version 2.1.0 (https://bioinfogp.cnb.csic.es/tools/venny/index.html) (Madrid, Spain, accessed on 19 September 2022). The predicted molecular weight (MW), theoretical isoelectric point (pI), and grand average of hydropathicity (GRAVY) of the SlCrRLK1Ls were determined by ExPASy-ProtParam (https://www.expasy.org/resources/protparam) (Lausanne, Switzerland, accessed on 19 September 2022) [63].

The obtained tomato, *Arabidopsis*, rice, and *Catharanthus roseus* CrRLK1L whole protein sequences were aligned by ClustalW and submitted to MEGA, version 11 (State College, PA, USA) [64], to construct a neighbor-joining phylogenetic tree with 1000 bootstrap replicates, a pairwise deletion, and a Poisson model. Then, the tree file was optimized by iTOL, version 6 (https://itol.embl.de/) (Heidelberg, Germany, accessed on 23 September 2022) [65].

### 4.2. Antibody Preparation

The specific polypeptide (KDLNESPGYDASMTDSRS) was synthesized and used as an antigen for immunizing rabbits in order to prepare the anti-SlCrRLK1L20 polyclonal antibody by the Abmart (Shanghai) company (Shanghai, China).

### 4.3. Western Blot Assay

The total proteins were extracted as described previously [30] and separated using a 10% SDS-PAGE gel. After the electrophoresis, the proteins were transferred to a PVDF membrane. The PVDF membrane was blocked in 5% skim milk for 1 h and then incubated with anti-HA (Abmart; 1:5000) and anti-SlCrRLK1L20 (this study; 1:1000) antibodies for 1 h, respectively. The images were captured by a chemiluminescent imaging system (Tanon). SlCrRLK1L20-HA and SlCrRLK1L20 were detected with the anti-HA and anti-SlCrRLK1L20 antibodies, respectively.

### 4.4. Gene Location and Collinearity Analysis

The location information of the *SlCrRLK1L* genes on the tomato chromosomes was obtained from the NCBI database and was illustrated by Advanced Circos (TBtools v1.108) [62]. The collinearity analysis of the *SlCrRLK1L* genes was conducted using the one-step MCScanX from TBtools with the default parameters [62].

### 4.5. Subcellular Localization Analysis

The SlCrRLK1L protein sequences were submitted to the Plant-mPLoc (http://www.csbio.sjtu.edu.cn/bioinf/plant-multi/) (Shanghai, China, accessed on 4 September 2022), CELLO, version 2.5, (http://cello.life.nctu.edu.tw/) (accessed on 4 September 2022) and MultiLoc2 (https://abi-services.informatik.uni-tuebingen.de/multiloc2/webloc.cgi) (Tübingen, Germany, accessed on 4 September 2022) webtools to predict their possible subcellular localization using the default parameters [66,67,68]. As for the subcellular localization of SlCrRLK1L20, a confocal assay was used. The SlCrRLK1L20 CDS was amplified by PCR and then inserted into pCAMBIA2300-GFP vectors. The recombinant plasmids were transferred into the *Agrobacterium tumefaciens* strain GV3101 and then infiltrated into the epidermal cells of *Nicotiana benthamiana*. The leaves were observed at 48 h post-infiltration by a laser scanning confocal microscope.

### 4.6. Protein Domain and Gene Structure Analyses

For the protein domain analyses, the SlCrRLK1L protein sequences were submitted to the NCBI Batch CD-Search (https://www.ncbi.nlm.nih.gov/Structure/bwrpsb/bwrpsb.cgi?) (Bethesda, MD, USA, accessed on 3 September 2022) [69] and processed using the default parameters, and the results (E-value < 1 × 10^−10^) were then obtained. The signal peptide and transmembrane helix regions were predicted by DeepTMHMM (https://dtu.biolib.com/DeepTMHMM) (Copenhagen, Denmark, accessed on 3 September 2022) [70] using the default parameters. The detailed protein domain data are listed in Table S4. For the gene structure analyses, the SlCrRLK1L gene annotation files were obtained from NCBI and subjected to the Visualize Gene Structure tools from TBtools for visualization.

### 4.7. Conserved Protein Motif Analysis

The SlCrRLK1L protein sequences were submitted to the MEME suite 5.5.0 webtool (https://meme-suite.org/meme/tools/meme) (San Diego, CA, USA, accessed on 1 September 2022) [71] and processed using the default parameters, and the result file was visualized using the Visualize MEME/MAST Motif Pattern (TBtools v1.108).

### 4.8. Promoter Analysis

The 2000 bp region upstream of the *SlCrRLK1L* CDS start sites was obtained from the tomato genome using GXF Sequence Extract (TBtools) and then submitted to PlantCARE (http://bioinformatics.psb.ugent.be/webtools/plantcare/html/) (Gent, Belgium, accessed on 3 September 2022) [72] and PlantTFDB, version 5.0 (http://planttfdb.gao-lab.org/index.php) (Beijing, China, accessed on 5 September 2022) [73], to identify the cis-acting elements and transcription factor binding sites using the default parameters. The results are listed in Tables S5 and S6 and visualized using the Simple BioSequence Viewer (TBtools v1.108).

### 4.9. Gene Expression Pattern Analysis

The *SlCrRLK1L* gene expression pattern analysis used RNA-seq data from the SGN tomato functional genomic database (SGN-TFGD, http://ted.bti.cornell.edu/cgi-bin/TFGD/digital/home.cgi) (Ithaca, NY, USA, accessed on 4 September 2022) [36,41]. For the expression pattern of the bacteria and the PAMP treatment, the expression data ratio was calculated and transformed with log2 to normalize. The data are listed in Tables S7 and S8. Morpheus (https://software.broadinstitute.org/morpheus/) (Cambridge, MA, USA, accessed on 4 September 2022) was adopted to illustrate the heatmap.

### 4.10. Accession Number

The detailed accession number can be found in Table S9.

## Figures and Tables

**Table 1:** List of the predicted CrRLK1L proteins in tomatoes.

Protein Name	Protein ID in NCBI	Protein Length (aa)	Molecular Weight (Da)	Theoretical pI	GRAVY	Matched SGN Locus	Annotation Difference in NCBI and SGN
SlCrRLK1L1	XP_025886195.1	854	95,039.67	6.86	−0.268	Solyc01g059910	Y
SlCrRLK1L2	XP_004230878.1	876	96,273.18	5.82	−0.212	Solyc01g109950	N
SlCrRLK1L3	XP_019067666.1	1152	129,597.94	6.93	−0.174	Solyc02g014030	N
SlCrRLK1L4	XP_004233885.1	868	96,438.64	5.49	−0.28	Solyc02g069970	Y
SlCrRLK1L5	XP_004233025.1	1002	112,230.67	6.36	−0.312	Solyc02g071860	Y
SlCrRLK1L6	XP_010316862.1	995	111,071.97	5.79	−0.108	Solyc02g071880	N
SlCrRLK1L7	XP_004232151.1	869	97,152.91	6.14	−0.234	Solyc02g089090	Y
SlCrRLK1L8	NP_001234869.1	903	101,461.32	8.92	−0.197	Solyc02g091590	N
SlCrRLK1L9	XP_004234657.2	817	91,323.59	5.9	−0.168	Solyc03g044160	Y
SlCrRLK1L10	XP_010318169.1	865	97,835	5.41	−0.201	Solyc03g093380	N
SlCrRLK1L11	XP_025886103.1	1340	150,300.63	6.39	−0.226	Solyc03g115710	Y
SlCrRLK1L12	XP_010318523.1	894	99,669.44	5.49	−0.16	Solyc03g121230	N
SlCrRLK1L13	XP_004239170.1	926	103,366.23	5.88	−0.267	Solyc05g014240	N
SlCrRLK1L14	XP_004240198.2	840	92,846.45	6.26	0.021	Solyc05g054680	N
SlCrRLK1L15	XP_004239762.1	811	90,461.22	5.66	−0.079	Solyc05g054860	N
SlCrRLK1L16	XP_004240568.1	887	97,458.64	5.73	−0.264	Solyc06g009540	N
SlCrRLK1L17	XP_004240569.1	880	97,075.96	6.33	−0.246	Solyc06g009550	Y
SlCrRLK1L18	XP_004243035.1	854	94,945.25	6.36	−0.259	Solyc07g008400	N
SlCrRLK1L19	XP_004246699.1	928	102,646.36	5.82	−0.212	Solyc09g007280	N
SlCrRLK1L20	XP_004246282.1	889	97,348.77	5.78	−0.22	Solyc09g015830	Y
SlCrRLK1L21	XP_004247083.1	904	101,257.01	5.48	−0.107	Solyc09g060110	N
SlCrRLK1L22	XP_010327142.1	840	91,947.64	5.26	0.032	Solyc10g006870	N
SlCrRLK1L23	XP_004248695.3	868	96,419.44	6.09	−0.221	Solyc10g054050	Y
SlCrRLK1L24	XP_004251295.1	836	91,944.54	5.84	−0.1	Solyc11g072910	Y

The putative tomato CrRLK1Ls are listed in Table 1. The protein length, molecular weight (MW), theoretical isoelectric point (pI), and grand average of hydropathicity (GRAVY) were analyzed. As shown in Table 1, the protein length ranged from 811 to 1340 aa, the MW ranged from 90,461.22 to 150,300.63 Da, the theoretical pI ranged from 5.26 to 8.92, and the GRAVY ranged from −0.312 to 0.032.

**Table 2:** The identified CrRLK1L numbers in various species.

Species	Number of CrRLK1Ls	Reference
*Arabidopsis thaliana*	17	[42]
*Oryza sativa* (rice)	16	[43]
*Malus domestica* (apple)	74	[40]
*Fragaria vesca* (strawberry)	62	[44]
*Citrus sinensis* ‘Valencia’ (Citrus)	47	[45]
*Glycine max* L. (soybean)	38	[33]
*Nicotiana tabacum* L. (tobacco)	48	[34]
*Nicotiana benthamiana*	31	[46]
*Chenopodium quinoa*	26	[35]
*Pyrus bretchneideri* (pear)	26	[32]
*Populus trichocarpa* (black cottonwood)	42	[47]
*Gossypium raimondii*, *G. arboreum*, and *G. hirsutum* TM-1 (cotton)	44	[48]
*Boea hygrometrica*	18	[49]
*Solanum tuberosum* (potato)	17	[50]
*Solanum lycopersicum* (tomato)	24	This study
*Amborella trichopoda*	9	[51]
*Marchantia polymorpha*	1	[52]
*Physcomitrella patens*	6	[53]
*Selaginella moellendorffii*	2	[54]
*Picea abies*	7	[55]
*Closterium peracerosum-strigosumlittorale* complex	1	[56]

## Data Availability

Not applicable.

## Supplementary Materials

The following supporting information can be downloaded at: https://www.mdpi.com/article/10.3390/ijms24043142/s1.
