# Supplementary material for: Genome-Wide Re-Identification and Analysis of CrRLK1Ls in Tomato
Source: Int J Mol Sci. 2023 Feb 5;24(4):3142. doi: 10.3390/ijms24043142 (PMC9959574; doi:10.3390/ijms24043142)
Supplement: Supplementary file 1 [file ijms-24-03142-s001.zip › Supplementary Materials-Figures.pdf]

Genome-wide re-identification and analysis of CrRLK1Ls in tomato

Wenpeng Ma [1], Xin Liu [1], Kai Chen [1], Xinlong Yu [1] and Dongchao Ji [2, 3, 4*]

[1] College of Agricultural Engineering and Food Science, Shandong University of Technology, Zibo 255049, China
[2] School of Life Sciences and Medicine, Shandong University of Technology, Zibo 255049, China; jidongchao@sdut.edu.cn
[3] Key Laboratory of Plant Resources, Institute of Botany, Innovative Academy of Seed Design, Chinese Academy of Sciences, Beijing 100093, China
[4] University of Chinese Academy of Sciences, Beijing 100049, China
* Correspondence: jidongchao@sdut.edu.cn

Supplementary Materials: The following supporting information can be downloaded at: www.mdpi.com/xxx/s1。

Figure S1: The tomato CrRLK1Ls gene structure in SGN.

Figure S2: Uncropped agarose gel and western blot images linked to Figure 2.

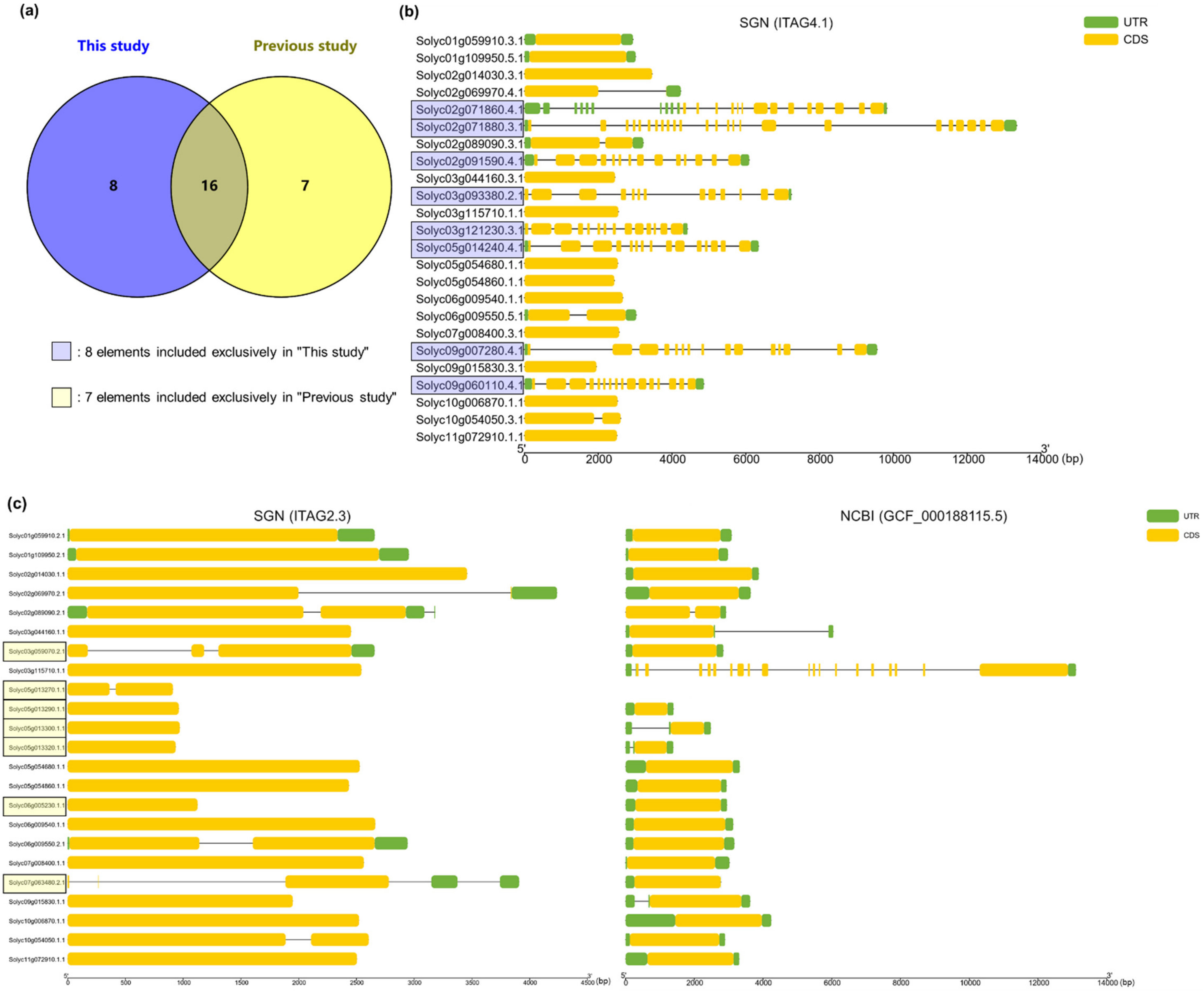

Figure S1. The tomato *CrRLK1Ls* gene structure in SGN. (**a**) The number of CrRLK1L members obtained in this study were compared with that obtained in previous study. (**b**) The tomato *CrRLK1Ls* gene structure in SGN (ITAG4.1). (**c**) The tomato *CrRLK1Ls* gene structure in SGN (ITAG2.3) and NCBI (GCF_000188115.5). The CrRLK1L members obtained in this study and previous study were marked in blue and yellow respectively. Data was extracted from SGN and NCBI, and then analyzed to visualize by TBtools.

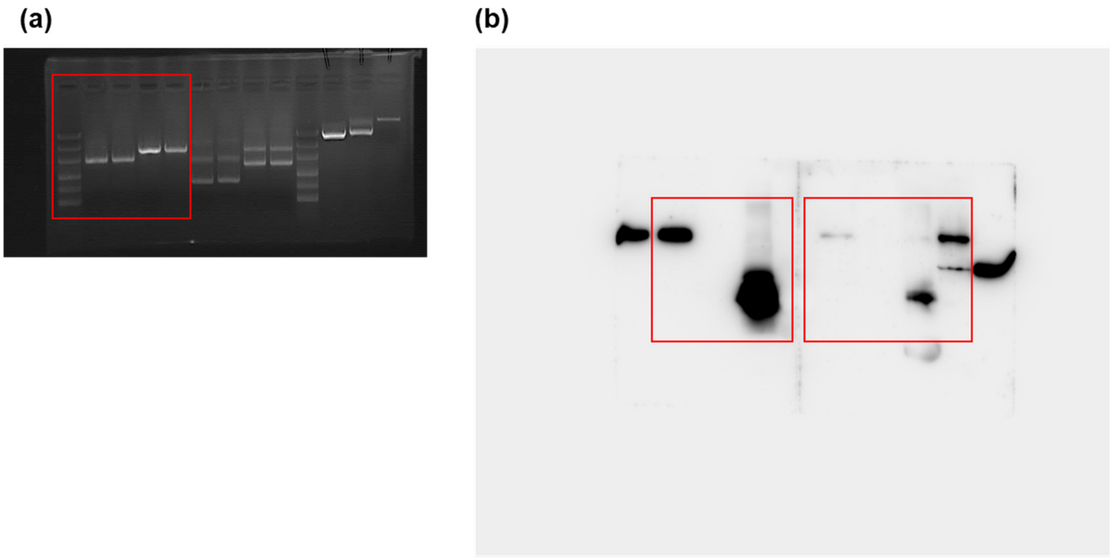

Figure S2. Uncropped agarose gel and western blot images linked to Figure 2. (**a**) Uncropped agarose gel for Figure 2(c); (**b**) Uncropped western blot images for Figure 2(d). Red lines represent the images used in Figure 2. Western blot with different antibodies were done respectively and chemiluminescent images were captured synchronously.
